# Seronegative Rheumatoid Arthritis in an Elderly Patient With Anemia: A Case Report

**DOI:** 10.7759/cureus.32239

**Published:** 2022-12-05

**Authors:** Kaho Aoe, Yuta Horinishi, Chiaki Sano, Ryuichi Ohta

**Affiliations:** 1 Gastroenterology, Himeji Red Cross Hospital, Himeji, JPN; 2 Community Care, Unnan City Hospital, Unnan, JPN; 3 Community Medicine Management, Faculty of Medicine, Shimane University, Izumo, JPN

**Keywords:** polyarthritis, anorexia, anemia of inflammation, seronegative rheumatoid arthritis, rural hospital, general medicine

## Abstract

Anemia due to chronic inflammation reduces the quality of life in the elderly population. Various causes of chronic inflammation exist, and the elderly experience varying symptoms, making it challenging to investigate the cause. The risk of chronic inflammatory diseases, including autoimmune diseases, and the risk of rheumatoid arthritis (RA) increase with age. Here, we report a case of seronegative RA in an 88-year-old woman who was referred for a detailed examination of chronic inflammation and anemia. Although she had no chief complaint, a physical examination revealed bilateral symmetric polyarthritis. After ruling out other diseases based on blood culture findings, the patient was diagnosed with seronegative RA. She was successfully treated with prednisolone and methotrexate, and her anemia improved. She also attributed the anemia to a chronic inflammatory pattern of seronegative RA. Appropriate physical examination is important for older adults with various complaints, and anemia may precede arthritis during the clinical course of RA. Additionally, inflammation may progress rapidly in these individuals.

## Introduction

There are various causes of anemia. In the elderly (those aged 65 years or older), iron, vitamin B1, and folic acid deficiencies account for one-third of anemia cases, and chronic inflammation and renal anemia account for another one-third of the cases. The remaining one-third of the cases have unknown causes [1]. Anemia associated with chronic inflammation is defined as elevated interleukin-6 levels that stimulate the production of hepcidin, which reduces intestinal iron absorption and circulating serum iron levels and increases total iron stores [1].

Management of underlying disease is essential for treating anemia associated with chronic inflammation [2]. Originally, this anemia was linked to chronic infections and autoimmune diseases, but in recent years, it has been found to be associated with other diseases, such as cancer and congestive heart failure [3].

We found polyarthritis on physical examination during a chronic inflammation workup. The differential diagnosis of polyarthritis in older patients includes collagen diseases, such as rheumatoid arthritis (RA), and infectious diseases, such as viral infections and infective endocarditis [4]. Joint aspiration and culture may help diagnose arthritis. Furthermore, arthritis is classified as acute/chronic, multiple/single, and pathological [5]. This case report discusses the relationship between RA and anemia and the factors causing anemia-associated inflammation to exacerbate rapidly enough to necessitate a blood transfusion.

## Case presentation

An 88-year-old woman with progressive anemia and chronic inflammation was referred to our hospital. Initially, she had a hemoglobin (Hb) level of 10 g/dL for 10 years. However, a month before her visit, the anemia rapidly declined her Hb level from 10 to 8 g/dL. Her medical history included hypertension, reflux esophagitis, insomnia, osteoporosis, dementia due to Alzheimer’s disease, and osteoarthritis in the knee. Her medications included losartan potassium, Benidipine, rabeprazole, denosumab, donepezil, and acetaminophen.

Her vital signs were as follows: blood pressure 147/67 mmHg; pulse rate 73 beats/minute; respiratory rate 16 breaths/minute; O_2_ saturation 96% in room air; and body temperature 36.9 °C. Physical examination revealed palpebral conjunctival anemia and no cervical lymphadenopathy. She exhibited normal breath sounds and a systolic heart murmur. Her extremities were warm and presented mild edema. Swelling and tenderness were observed in the right fourth, left third, and left fourth finger metacarpophalangeal joints and in the elbow, knee, and ankle joints. The blood test results show severe anemia and high inflammatory condition, with an Hb level of 8.0 g/dL, erythrocyte sedimentation rate (ESR) of 124 mm, and C-reactive protein (CRP) level of 16.18 mg/dL (Table 1).

**Table 1:** Initial laboratory data of the patient. eGFR, estimated glomerular filtration rate; CK, creatine kinase; CRP, C-reactive protein; Ig, immunoglobulin; C3, complement component 3; C4, complement component 4; KL-6, Krebs von den Lungen-6; MPO-ANCA, myeloperoxidase-antineutrophil cytoplasmic antibody; PR3-ANCA, proteinase-3-antineutrophil cytoplasmic antibody; CCP, cyclic citrullinated peptide; MMP-3, matrix metalloproteinase-3

Marker	Value	Reference
White blood cell count	5.50 × 10^3^ μL^-1^	3.5 × 10^3^ to 9.1 × 10^3^ μL^-1^
Neutrophil differential count	74.6%	44.0%-72.0%
Lymphocyte differential count	16.2%	18.0%-59.0%
Monocyte differential count	7.9%	0.0%-12.0%
Eosinophil differential count	0.3%	0.0%-10.0%
Basophil differential count	1.0%	0.0%-3.0%
Red blood cell count	2.72 × 10^6^ μL^-1^	3.76 × 10^6^ to 5.50 × 10^6^ μL^-1^
Hemoglobin level	8 g/dL	11.3-15.2 g/dL
Hematocrit	24.1%	33.4%-44.9%
Mean corpuscular volume	88.6 fL	79-100 fL
Platelet count	50.4 × 10^4^ μL^-1^	13.0 × 10^4^ to 36.9 × 10^4^ μL^-1^
Erythrocyte sedimentation rate	124 mm	3-15 mm
Total protein level	7.7 g/dL	6.5-8.3 g/dL
Albumin level	3.0 g/dL	3.8-5.3 g/dL
Total bilirubin level	0.4 mg/dL	0.2-1.2 mg/dL
Aspartate aminotransferase level	16 IU/L	8-38 IU/L
Alanine aminotransferase level	10 IU/L	4-43 IU/L
Alkaline phosphatase level	104 U/L	106-322 U/L
γ-Glutamyl transpeptidase level	25 IU/L	<48 IU/L
Lactate dehydrogenase level	285 U/L	121-245 U/L
Blood urea nitrogen level	5.5 mg/dL	8-20 mg/dL
Creatinine level	0.86 mg/dL	0.40-1.10 mg/dL
eGFR	46.7 mL/min/L	>60 mL/min/L
Serum sodium level	137 mEq/L	135-150 mEq/L
Serum potassium level	4.0 mEq/L	3.5-5.3 mEq/L
Serum chloride level	102 mEq/L	98-110 mEq/L
Serum ferrum level	14 µg/dL	43-172 µg/dL
Ferritin level	435.6 ng/mL	14.4-303.7 ng/mL
CK level	73 U/L	56-244 U/L
CRP level	16.18 mg/dL	<0.30 mg/dL
IgG level	2,095 mg/dL	870-1,700 mg/dL
Antinuclear antibody level	<40	<40
Homogeneous	<40	<40
Speckled	<40	<40
Nucleolar	<40	<40
Peripheral	<40	<40
Discrete SP	<40	<40
Cytoplasm	<40	<40
C3 level	161 mg/dL	86-160 mg/dL
C4 level	32 mg/mL	17-45 mg/mL
KL-6 level	178 U/mL	105.3-401.2 U/mL
MPO-ANCA level	<1.0 U/mL	<3.5 U/mL
PR3-ANCA level	<1.0 U/mL	<3.5 U/mL
Rheumatoid factor level	0 IU/mL	<15 IU/mL
CCP antibody level	<0.6 U/mL	<5 U/mL
MMP-3 level	≥1,600 ng/mL	17.3–59.7 ng/mL

Contrast-enhanced CT of the abdomen and pelvis revealed no obvious active bleeding, and upper and lower endoscopy revealed several polyps in the large intestine. No neoplastic lesions or lesions that could cause bleeding were observed. A gynecological examination revealed no bleeding lesions. The X-ray revealed no narrowing of the joint space or bone erosion of the hands, elbows, feet, or knees. Joint ultrasonography revealed synovial thickening and increased blood flow to the wrist, elbow, knee, and ankle joints.

The patient's laboratory data showed normocytic anemia. However, there was no apparent source of bleeding. The patient also exhibited high ferritin levels and ESR values. We determined that her anemia was associated with chronic inflammation. In the search for chronic inflammation, we observed swelling and pain in the joints, including in the bilaterally symmetrical small joints. The results for rheumatoid factor and anticitrullinated peptide antibody (ACPA) tests were negative. However, the patient’s matrix metalloproteinase-3 levels were markedly increased. Joint echography revealed synovial hyperplasia and increased blood flow. At this point, RA Class 4 was determined because of the involvement of four small joints, elbows, knees, and ankles; negative serologic tests; high CRP levels; ESR; and the unknown time of the onset of symptoms [5]. Blood culture results were negative. Cancer and other connective tissue diseases were unlikely. Although these criteria were not met, we temporarily diagnosed the patient with seronegative RA. The Disease Activity Score (DAS)-CRP was 5.89, showing high activity at the start of treatment.

Methotrexate (6 mg/week) and prednisolone (10 mg) were started on Day 6. After the treatment, the joints' swelling and tenderness gradually improved. Blood tests on Day 12 revealed blood sedimentation of 70 mm and a CRP level of 1.41 mg/dL. The DAS-CRP score improved to 3.34. We prioritized the treatment of the primary disease of seronegative RA. The patient started oral iron preparations on Day 4 of admission. On Day 8 of admission, anemia worsened, and the Hb level decreased to 6.4 g/dL without accompanying symptoms such as light-headedness or palpitations. Blood transfusions are still the only feasible option for the elderly with severe, symptomatic anemia [2]. Regarding recommendations about transfusions in the elderly, two units of red blood cells were transfused because Hb was less than 7.0 on Day 8 of admission [6]. Her arthritic symptoms and anemia improved to 10.4 g/dL on Day 16 of admission, and she was discharged on Day 18. In outpatient follow-up, her anemia improved to 11 g/dL without blood transfusion and any flair of RA.

## Discussion

This case report is about an 88-year-old woman referred for a detailed examination of chronic inflammation and anemia. She presented with bilateral symmetric polyarthritis, and after ruling out other diseases by performing blood culture tests, she was diagnosed with seronegative RA. Her anemia demonstrated a chronic inflammatory pattern, possibly caused by seronegative RA. This case report reviews the relationship between arthritis and anemia and the factors contributing to the acute progression of anemia associated with chronic inflammation.

To investigate the relationship between arthritis and anemia, we searched for cases in which anemia preceded arthritis in patients with RA to investigate which precedes first. We searched PubMed using the terms “rheumatoid arthritis,” “anemia,” and “preceding arthritis” on September 2, 2022. However, there were no case reports identified in the search. Joint symptoms are positively correlated with anemia. Han et al. reported that joint damage is more pronounced in patients with lower Hb levels [6]. Even after adjusting for disease activation in DAS28 and disease duration, similar results were observed [6]. Wilson et al. reported that patients with RA and anemia have more severe joint symptoms, such as joint swelling, pain, and tenderness [7]. However, when anemia is successfully treated, joint symptoms are likely to improve from the treatment [7]. The patient had progressive anemia and arthritis but no spontaneous complaints of joint pain. At the initial presentation, she appeared to have anemia preceding arthritis. However, she may not have complained of arthralgia due to Alzheimer’s dementia. The patient reported no symptoms. Therefore, the actual onset time and order in which the symptoms appeared are unclear. When we started treatment, anemia and joint tenderness improved, and the clinical course was similar to that in previous reports [6,7].

Next, we discussed why anemia caused by chronic inflammation was rapidly exacerbated. Inflammation-induced anemia is the second most common anemia worldwide after iron deficiency anemia [8]. As many as 40% of all anemia cases worldwide are inflammation-related [8]. Its progression is generally slow, and the reduction in the Hb level remains mild to moderate [8]. In another study, 31.5% of patients with RA had anemia, but severe chronic anemia (Hb level < 10 g/dL) was rare (3.4%) [9]. The optimal treatment for anemia of inflammation is to treat the underlying inflammatory disease, which improves in most cases [9]. However, 20%-85% of cases are associated with iron deficiency anemia, and distinguishing between the two is occasionally difficult [8]. Therefore, iron supplementation and erythropoiesis-stimulating agents are often used to treat anemia caused by inflammation [8]. The symptoms of anemia improved with therapy for RA, which is the primary disease, and the oral administration of iron preparations was initiated.

There are possible reasons why her anemia exacerbated so rapidly that a blood transfusion was temporarily required. In previous reports, the refeeding syndrome caused hemolytic anemia when dietary intake was started owing to a lack of electrolytes and an inability to shift metabolic demands to increase red blood cell production [10]. She had anorexia one month before admission but consumed food orally after hospitalization. As a result, the patient may have developed the refeeding syndrome. The balance between erythropoiesis and hemolysis collapsed, and her anemia temporarily worsened.

Inflammation-induced anemia can be improved by treating the underlying disease. This should be investigated by family physicians in rural settings. Anemia in older patients can be missed in modern clinical medicine because of ageism, particularly in rural contexts [11-13]. Moreover, older patients’ symptoms related to anemia may be vague and do not effectively trigger their help-seeking behaviors [14,15]. Therefore, suspicion of anemia of inflammation among older patients with vague symptoms from data, such as blood tests, and identification of the underlying disease are important. Inflammation generally progresses slowly, and the reduction in Hb levels is rarely severe [8]. However, older adults and patients with dementia may develop severe anemia without any subjective symptoms such as pain [16,17]. Older patients with vague symptoms require comprehensive care using systemic approaches in rural contexts [18]. Family physicians are specialists in both systems and health care. They should be keen to comprehensively examine older patients’ symptoms and continuously promote their health conditions continuously [19,20].

## Conclusions

We report a case of seronegative RA in an elderly patient diagnosed with anemia. The treatment of RA with methotrexate and prednisolone improved her anemia. Inflammation can be improved by treating the underlying disease. However, if the nutritional status is poor before the start of treatment, anemia may temporarily worsen. Older adults and patients with dementia may not complain of symptoms, and anemia may be aggravated without being noticed. More attention should be paid to physical examination.

## References

[1] Guralnik JM, Eisenstaedt RS, Ferrucci L, Klein HG, Woodman RC (2004). Prevalence of anemia in persons 65 years and older in the United States: evidence for a high rate of unexplained anemia. Blood.

[2] Girelli D, Marchi G, Camaschella C (2018). Anemia in the elderly. Hemasphere.

[3] Nikiphorou E, Santos EJ, Marques A (2021). 2021 EULAR recommendations for the implementation of self-management strategies in patients with inflammatory arthritis. Ann Rheum Dis.

[4] Kojima M, Nakayama T, Tsutani K (2020). Epidemiological characteristics of rheumatoid arthritis in Japan: prevalence estimates using a nationwide population-based questionnaire survey. Mod Rheumatol.

[5] Aletaha D, Smolen JS (2018). Diagnosis and management of rheumatoid arthritis: a review. JAMA.

[6] Han C, Rahman MU, Doyle MK (2007). Association of anemia and physical disability among patients with rheumatoid arthritis. J Rheumatol.

[7] Wilson A, Yu HT, Goodnough LT, Nissenson AR (2004). Prevalence and outcomes of anemia in rheumatoid arthritis: a systematic review of the literature. Am J Med.

[8] Weiss G, Ganz T, Goodnough LT (2019). Anemia of inflammation. Blood.

[9] Wolfe F, Michaud K (2006). Anemia and renal function in patients with rheumatoid arthritis. J Rheumatol.

[10] Byrnes MC, Stangenes J (2011). Refeeding in the ICU: an adult and pediatric problem. Curr Opin Clin Nutr Metab Care.

[11] Amano S, Ohta R, Sano C (2021). Recognition of anemia in elderly people in a rural community hospital. Int J Environ Res Public Health.

[12] Horinishi Y, Shimizu K, Sano C, Ohta R (2022). Surgical interventions in cases of esophageal hiatal hernias among older Japanese adults: a systematic review. Medicina (Kaunas).

[13] São José JM, Amado CA (2017). On studying ageism in long-term care: a systematic review of the literature. Int Psychogeriatr.

[14] Ohta R, Ryu Y, Sano C (2022). Older people's help-seeking behaviors in rural contexts: a systematic review. Int J Environ Res Public Health.

[15] Ohta R, Ryu Y, Kitayuguchi J, Sano C, Könings KD (2021). Educational intervention to improve citizen's healthcare participation perception in rural Japanese communities: a pilot study. Int J Environ Res Public Health.

[16] Yamashita M, Ohta R, Mouri N, Takizawa S, Sano C (2022). Herpes simplex virus pneumonia mimicking Legionella pneumonia in an elderly patient with heart and liver failure. Cureus.

[17] Tokonami A, Ohta R, Katagiri N, Yoshioka N, Yamane F, Sano C (2022). Autoimmune vasculitis causing acute bilateral lower limb paralysis. Cureus.

[18] Kusunoki M, Ohta R, Suzuki K, Maki T, Sano C (2022). Inquiry into physicians' scope of practice in Japanese rural hospitals during the COVID-19 pandemic: a serial cross-sectional study. Cureus.

[19] Ohta R, Sano C (2022). Family physicians as system-specific specialists in Japan's aging society. Cureus.

[20] Ohta R, Sano C (2022). Implementation of the principles of family medicine in modern family medicine education needing system-specific approaches. Cureus.

